# Sclerostin antibody improves alveolar bone quality in the *Hyp* mouse model of X-Linked Hypophosphatemia (XLH)

**DOI:** 10.21203/rs.3.rs-2762671/v1

**Published:** 2023-04-12

**Authors:** Ryan Ross, Kelsey Carpenter, Delia Alkhatib, Bryan Dulion, Elizabeth Guirado, Shreya Patel, Yinghua Chen, Anne George

**Affiliations:** Rush University Medical Center; Rush University Medical Center; Rush University Medical Center; Rush University Medical Center; UIC; Rush University Medical Center; University of Illinois Chicago College of Dentistry; Department of Oral Biology

**Keywords:** Rickets, hypophosphatemia, osteomalacia, periodontium, mineralized tissue/development

## Abstract

X-linked hypophosphatemia (XLH) is a rare disease of elevated fibroblast growth factor 23 (FGF23) production that leads to hypophosphatemia and poor mineralization of bone and teeth. The clinical manifestations of XLH include a high prevalence of dental abscesses, likely driven by poorly formed structures of the dentoalveolar complex, including the alveolar bone, cementum, dentin, and periodontal ligament. Our previous studies have demonstrated that sclerostin antibody (Scl-Ab) treatment improves phosphate homeostasis, and increases bone mass, strength and mineralization in the *Hyp* mouse model of XLH. In the current study, we investigated whether Scl-Ab impacts the dentoalveolar structures of *Hyp* mice. Male and female wild-type and *Hyp* littermates were injected with 25 mg/kg of vehicle or Scl-Ab twice weekly beginning at 12 weeks of age and euthanized at 20 weeks of age. Scl-Ab increased alveolar bone mass in both male and female mice and alveolar tissue mineral density in the male mice. The positive effects of Scl-Ab were consistent with an increase in the fraction of active (non-phosphorylated) β-catenin stained alveolar osteocytes. Scl-Ab had no effect on mineralized tissues of the tooth - dentin, enamel, acellular and cellular cementum. There was a non-significant trend toward increased periodontal ligament (PDL) attachment fraction within the *Hyp* mice. Additional PDL fibral structural parameters were not affected by Scl-Ab. The current study demonstrates that Scl-Ab can improve alveolar bone in the *Hyp* mouse model of XLH.

## Introduction

X-linked hypophosphatemia (XLH) is an inherited rickets caused by inactivating mutations in phosphate regulating neutral endopeptidase on the X-chromosome (PHEX). Loss of PHEX results in elevated fibroblast growth factor 23 (FGF23), a circulating phosphaturic hormone that acts on the kidney to decrease phosphate reabsorption and inhibit 1,25-dihydroxyvitamin D production^1^. The resulting hypophosphatemia contributes to poor mineralization of both bone and teeth.

Between 50–80% of XLH patients deal with dental complications^2,3^, including tooth fractures, abscesses, and periodontal disorders^3,4^. Abnormalities in the matrix of dentoalveolar tissues, including impaired mineralization and large interglobular spaces^5^ likely contribute to recurrent dental abscesses and the high prevalence of periodontitis. A more detailed description of the dentoalveolar tissue pathologies associated with loss of PHEX function has been obtained using the *Hyp* mouse model of XLH. *Hyp* mice present with porous alveolar bone, enlarged pulp space, thin acellular cementum, hypomineralized cellular cementum, and detachment of the periodontal ligament (PDL)^6,7^.

Sclerostin is a circulating antagonist to Wnt signaling. Sclerostin null mice have increased bone mass driven by elevated bone formation^8^. In the dentoalveolar compartment, sclerostin null mice have increased alveolar bone mass and acellular and cellular cementum thickness, as well as decreased PDL space^9^. The gene encoding sclerostin (SOST) is primarily produced by bone embedded osteocytes^10^, as well as by cementocytes, particularly during the late stages of cementum development^11,12^. Sclerostin is also expressed by cells within the PDL, both in vivo and in vitro, and its expression is significantly upregulated in response to mechanical loads^13^.

Our previous research found that sclerostin antibody (Scl-Ab) increases bone mass, decreases FGF23, and increases phosphate in growing and adult *Hyp* mice^14,15^. The goal of this study is to evaluate the efficacy of Scl-Ab in improving the dentoalveolar defects in *Hyp* mice. We hypothesized that Scl-Ab treated *Hyp* mice will have increased alveolar bone volume, dentin/cementum volume, cellular cementum area, and PDL attachment.

## Results

### Scl-Ab improves alveolar bone mass in both *Hyp* and WT mice

Previous characterization of these mice found that Scl-Ab reduces FGF23 levels and increases trabecular bone mass in the distal femoral metaphysis and cortical bone mass in the femoral midshaft^14^.

**Figure 1:**
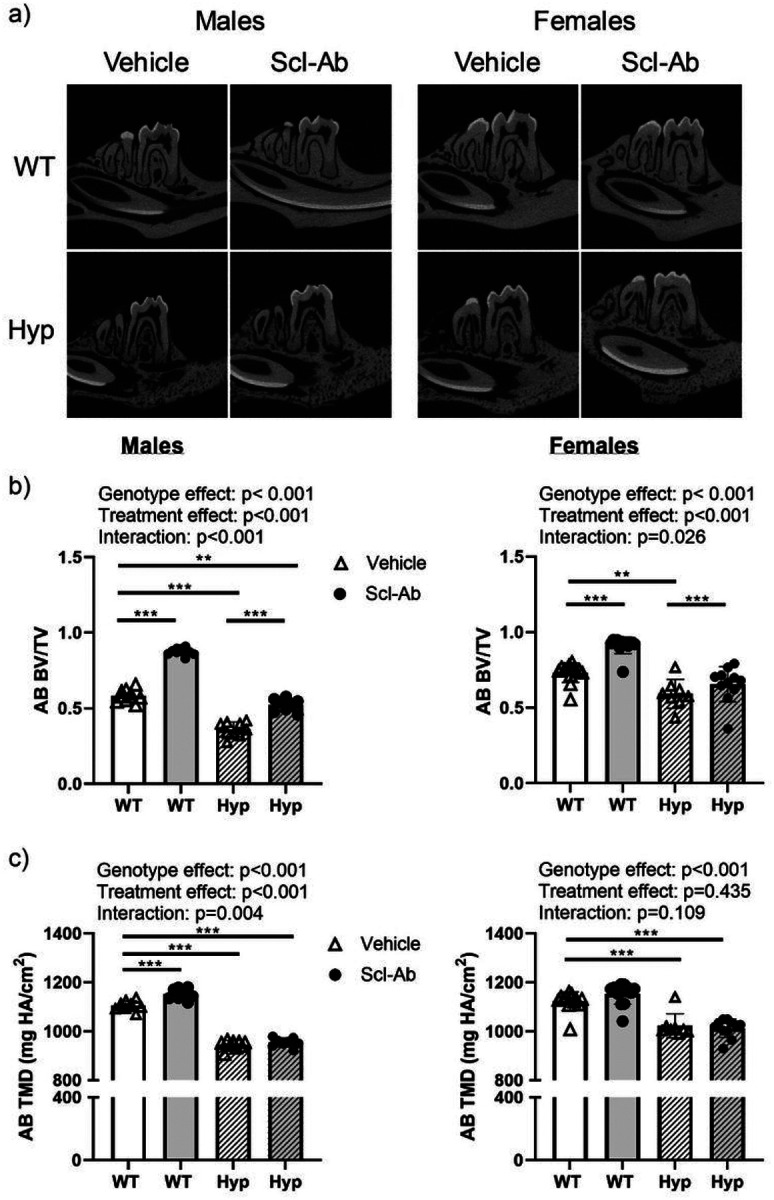
Representative sagittal microCT images of hemi-mandibles (top). (b) Alveolar bone volume per total volume and (c) tissue mineral density in male (left) and female (right) mice. Data are presented as the mean ± standard deviation. Results from the two-way analysis of variance (ANOVA) are presented in the figure legends. Significant post-hoc treatment differences between animals of the same genotype are presented as a horizontal bar. P-value thresholds are indicated with stars above the bars: *p<0.05; **p<0.01; ***p<0.001; ****p<0.0001.

*Hyp* alveolar bone presented with decreased alveolar bone volume per total volume (AB BV/TV) when compared WT mice in both sexes (Fig. 1 & Supplemental Table 1). Scl-Ab increased AB BV/TV regardless of genotype or sex (Fig. 1). Overall, WT mice exhibited greater increases in AB BV/TV when compared to *Hyp* mice (males: 50% WT, 45% *Hyp*; females: 25% WT, 11% *Hyp*).

Increased AB BV/TV following Scl-Ab treatment was primarily due to increased alveolar bone volume (Supplemental Table 1). Scl-Ab increased alveolar bone volume regardless of genotype or sex, although the post-hoc comparisons were not significant in the *Hyp* female mice.

By contrast, increases in alveolar tissue mineral density (AB TMD) in response to Scl-Ab were only observed in male mice. There was also a significant genotype-by-treatment interaction in male mice, attributed to the significant increase in WTs but a lack of a post-hoc difference in *Hyp* mice. No significant differences in alveolar tissue mineral density were observed in female mice.

**Figure 2:**
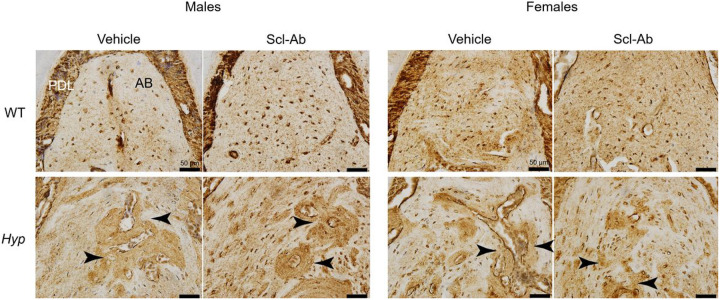
Osteopontin immunostaining of alveolar bone in male (left) and female (right) WT and Hyp mice treated with vehicle or Scl-Ab. Staining was performed within the alveolar bone (AB) between the tooth roots of the first molar (M1). Arrows point to dense regions of OPN staining within the alveolar bone of *Hyp* mice, AB labels the alveolar bone and PDL labels the periodontal ligament connecting the tooth roots and the alveolar bone.

**Figure 3:**
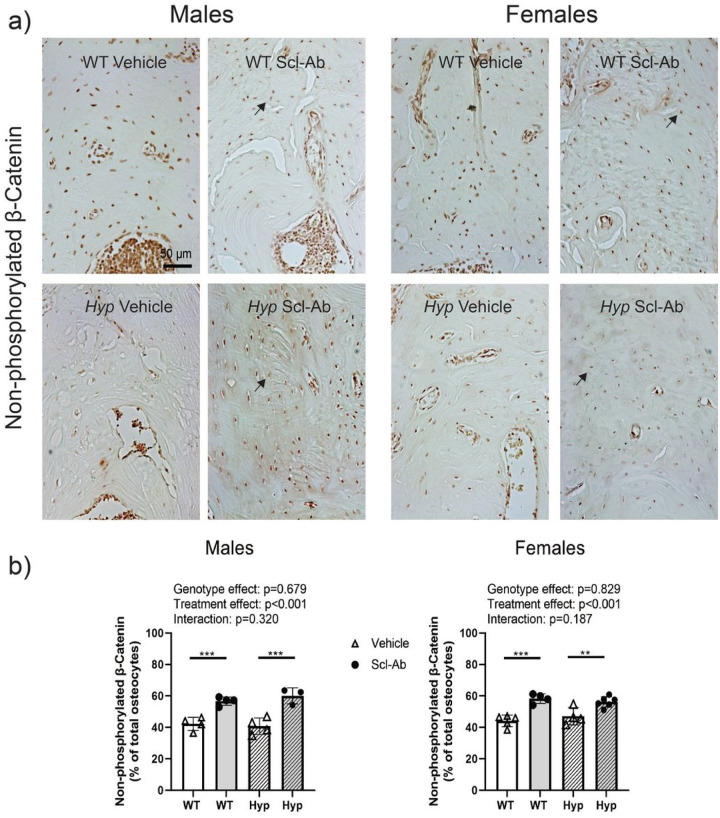
Non-phosphorylated β-catenin immunostaining in the alveolar bone between the tooth roots of the first molar in male (left) and female (right) WT and *Hyp* mice treated with vehicle or Scl-Ab. (a) Representative images of alveolar bone regions showing osteocytes embedded within the bone matrix. Single arrows highlight positively stained embedded osteocytes, while double arrows highlight positively stained extracellular bone matrix. (b) Quantitative measurements of the fraction of non-phosphorylated β-catenin stained osteocytes normalized by the total number of bone-embedded osteocytes within the alveolar bone region of interest. Data are presented as the mean ± standard deviation. Results from the two-way analysis of variance (ANOVA) are presented in the figure legends. Significant post-hoc treatment differences between animals of the same genotype are presented as a horizontal bar. P-value thresholds are indicated with stars above the bars: *p<0.05; **p<0.01; ***p<0.001.

Male and female *Hyp* mice present with regions of high osteopontin (OPN) staining within the alveolar bone (Fig. 2, black arrows), whereas the WT animals present with lower OPN staining and a more diffuse staining pattern. Scl-Ab did not affect the distribution of OPN of *Hyp* mice of either sex (Fig. 2). Non-phosphorylated β-catenin staining was used to evaluate the activation of Wnt signaling within osteocytes. Following Scl-Ab treatment, more osteocytes within the alveolar bone stained positively for non-phosphorylated β-catenin (Fig. 3). The number of alveolar bone osteocytes positively stained relative to the total number of osteocytes demonstrated a significant treatment effect (p < 0.001 for both male and female mice). Specifically, Scl-Ab treated mice had an increased percentage of positively stained osteocytes within the alveolar bone when compared to vehicle treated animals of both genotypes. DMP1 was used as marker of osteoblast differentiation^16^. The staining shows increases in both the number of positively stained osteocytes (Supplemental Fig. 1, single arrows) and in extracellular matrix staining (double arrows) following Scl-Ab treatment in both WT and *Hyp* mice, consistent with the role of DMP1 in mineralization as well.

### Scl-Ab had no effect on tooth structures

Male and female *Hyp* mice have significantly decreased enamel volume compared to WT mice (genotype effect: p = 0.001, both sexes, Supplemental Table 1). Scl-Ab did not affect the enamel volume in either WT or *Hyp* mice. Similarly, the dentin/cementum volume was significantly decreased in *Hyp* compared to WT mice (genotype effect: p < 0.001, both sexes) and Scl-Ab did not affect dentin/cementum volume in either genotype. Male and female *Hyp* mice have significantly increased pulp volume when compared to WT mice (genotype effect: p < 0.001, both sexes). Although there were no Scl-Ab treatment effects in either genotype, there was a significant genotype-treatment interaction in female mice (p = 0.043), with Scl-Ab increasing and decreasing pulp volume in WT and *Hyp* mice, respectively. Enamel and dentin/cementum tissue mineral density were not affected by either genotype or treatment.

The acellular cementum layer was significantly thinner in *Hyp* mice of both sexes (genotype effect: p < 0.001, both males and females, Table 1). Scl-Ab did not affect the acellular cementum thickness in either WT or *Hyp* mice. The pre-dentin layer was significantly thicker in *Hyp* mice of both sexes (genotype effect: p < 0.001 and 0.018, males and females, respectively, Table 1), but there was no treatment effect in either WT or *Hyp* mice. The cellular cementum area was affected by neither genotype nor Scl-Ab treatment (Table 1).

### Scl-Ab had limited effects on the PDL

The fraction of tooth root in direct contact with PDL fibers was significantly decreased in *Hyp* mice when compared to WTs (genotype effect: p < 0.001, both sexes, Table 2). Scl-Ab did not significantly affect the PDL attachment fraction, likely due to the high variability in *Hyp* samples and the fact that WTs were already at nearly 100% attachment (Table 2 & Supplemental Fig. 2).

Polarized imaging was used to evaluate PDL fiber angle, length, and width. The quantitation of polarized images using CT-FIRE failed to detect significant genotype or treatment effects in the fiber angle, length or width parameters (Table 2 & Supplemental Fig. 2).

## Discussion

Dental complications are a common in adults with XLH, with between 50–80% of patients reporting oral health complaints^2,3^. While novel treatment strategies, such as anti-FGF23 antibody (Burosumab), have been effective in improving skeletal and growth-related pathologies, there were initial reports that the number of dental abscesses is greater with Burosumab treatment when compared to conventional phosphate supplementation^17^. More recent data has suggested that if initiated early in children, Burosumab can significantly reduce the number of abscesses when compared to phosphate treatment^18,19^. The current study was undertaken to understand whether sclerostin contributes to the dentoalveolar defects and to evaluate the use of sclerostin antibody (Scl-Ab) in the *Hyp* mouse model of XLH. Importantly, poor alveolar bone mass and mineralization likely contribute to the high rates of dental abscesses and excessive tooth movement in XLH patients, and our results demonstrate that Scl-Ab treatment significantly improves alveolar bone mass, without affecting other tissues within the dentoalveolar complex.

The *Hyp* mouse is a well-characterized preclinical model of XLH that features the same elevated FGF23, hypophosphatemia, osteomalacia and low bone mass noted in XLH^20^. Additionally, *Hyp* mice present with the same decreased mass and mineralization of dentoalveolar tissues^6,21^. The defects noted in the alveolar bone, cementum, and periodontal ligament likely contribute to an increase risk of tooth loss^4^. Additionally, poor alveolar bone quality likely contributes to the high rates of implant failures reported in XLH patients^3^.

Currently, treatment strategies for XLH aim to improve phosphate levels with phosphate and vitamin D supplementation or, more recently, by targeting FGF23 with neutralizing antibodies. While both strategies can improve skeletal pathologies, they appear to differ in their effects on periodontal tissues. Lira Dos Santos et al. compared the effects of vitamin D and FGF23 antibody treatment in *Hyp* mice and noted that while both treatments increased phosphate levels, vitamin D was more effective than FGF23 antibody at improving alveolar bone and acellular cementum thickness^22^. A finding that appears consistent with clinical observations, wherein vitamin D appears to reduce the burden of oral infections^4^. Early data with the FGF23 antibody found increased dental abscesses following treatment^17^, but more recent data suggests that children treated early with Burosumab have reduced dental abscesses^18,19^

Our previous publications have demonstrated the positive effects of Scl-Ab treatment on FGF23 and phosphate levels, as well as, bone mass, mineralization and strength in *Hyp* mice^14,15^. The current study expands these findings to show that Scl-Ab has positive effects on alveolar bone in *Hyp* mice, perhaps unsurprisingly, as osteocytes within the alveolar bone are known to express sclerostin protein^10,12^. Interestingly, cementocytes within the cellular cementum and cells within the PDL also express sclerostin^11–13^ and yet Scl-Ab had little to no effect on these tissues. It is unclear why the non-bone tissues are not as responsive to Scl-Ab treatment. It is possible that the relatively late stage of development evaluated in this study – near the end of skeletal maturity and past molar eruption – contributed to the limited effects outside of bone. Additionally, while bone, cellular cementum, and PDL are all capable of cellular-mediated tissue repair, in the cellular and the PDL this repair is primarily reported in response to tooth movement forces^23,24^. Therefore, it is possible that without external forces, cementum and the PDL are less likely to respond to Scl-Ab. We designed the experiment to test the effects of Scl-Ab in adult mice, as dental complications are a common complaint in adult XLH patients. However, it is worth noting that clinical data have suggested that early and sustained treatment starting in childhood had the most significant impact on preventing periodontitis in XLH patients^4^. Indeed, new data reported that children with XLH that receive Burosumab before they reach 5 years of age had fewer dental abscesses than children treated with conventional phosphate supplementation, but children treated after 5 years of age did not see the same benefit^19^. Therefore, future work should aim to evaluate whether early Scl-Ab treatment would have a more significant benefit on periodontal pathologies.

Similar positive effects of Scl-Ab on alveolar bone were in the DMP1 null mouse model of autosomal recessive hypophosphatemic rickets (ARHR)^25^. However, unlike the current study, the authors report that Scl-Ab improved the cellular cementum and PDL organization, although it is worth noting that these effects were not compared quantitatively, as in the current study. Interestingly, Scl-Ab had no effect on circulating FGF23 in the ARHR mouse^25^, unlike in the *Hyp* mouse, where we previously reported decreased FGF23 following Scl-Ab treatment^14,15^. The positive effects in the alveolar bone of both the ARHR and *Hyp* mouse models suggest that these are likely a direct effect of sclerostin suppression, rather than a secondary effect of improved phosphate metabolism. However, directly assessing the relative contributions of tissue level and systemic changes is difficult. Indeed, although data from the sclerostin knockout mouse has demonstrated increased alveolar bone mass and increased cementum thickness^9^, systemic characterization of mineral metabolism in this mouse has found reduced FGF23 and increased vitamin D when compared to WT littermates^26^. Therefore, we cannot rule out that the Scl-Ab mediated suppression of circulating FGF23 also contributes to improve matrix mineralization.

Despite increased alveolar bone material density via microCT, the OPN staining failed to demonstrate a Scl-Ab treatment effect. Abnormal OPN accumulation, in the form of dense staining around osteocytic lacunae, has previously been described in the alveolar bone, calvaria, and tibia of *Hyp* mice and has been proposed as a mineralization inhibitor^22,27^. Indeed, genetically ablating OPN in *Hyp* mice reduced unmineralized osteoid accumulation in long bones^28^. While the alveolar bone material density would suggest a reduction in osteoid, a limitation of this study is that we did not have mineralized tissue to directly measure osteoid area. Our previous studies found a minor decrease in osteoid following Scl-Ab treatment in the long bones of *Hyp* mice^14,15^, but it is unclear whether this same effect occurs in alveolar bone.

Although direct sex comparisons were not part of our study design, we evaluated both male and female *Hyp* mice. Scl-Ab positively affected alveolar bone mass in both, but the magnitude change appears to be greater in males, while statistically significant changes in alveolar bone TMD were only present in males. It is unclear whether these differences are due to the X-linked nature of the disease or sex-specific responses to Scl-Ab. XLH is an autosomal dominant disease and therefore affects both males and females^29^. Clinically, there does not appear to be biochemical differences between male and female XLH patients^30^. While, there have been reports that height reduction is more significant in males^31^, a composite endodontic score that included a measure of periodontitis failed to find sex differences^31^.Clinically, Scl-Ab is approved for the treatment of postmenopausal women and few studies have been performed in men. One clinical safety study that did include both found that both had increased bone mass but did not directly compare between sexes^32^. A direct sex comparison of Scl-Ab treatment was performed in a mouse model of osteogenesis imperfecta and reported that some skeletal changes were greater in male mic^33^, consistent with the current study. Therefore, it seems likely that sclerostin suppression may affect males more than females, but the cause for these sex-specific responses is unknown.

The clinical implications of the current study remain to be determined. Although the effect of Scl-Ab on additional dentoalveolar tissues were limited, clinical reports have noted general alveolar bone loss as a contributing factor to tooth movement in XLH patients^34^. Therefore, improving alveolar bone quality would likely improve oral health for XLH patients, however, future work evaluating tooth mobility, tooth abscess development, or the stability of dental implants following Scl-Ab treatment is needed. One limitation worth noting is that our alveolar bone measurements were made between the tooth roots, or the bone within the furcation. Although this likely reflects general alveolar bone response, cervically located alveolar bone is a critical component of tooth stability and is not reflected in our alveolar bone measurements. We also studied a single Scl-Ab dose, however, the dose use is commonly used in rodent studies^35^ and bone anabolic responses have been noted in doses less than 1/10 of the one used here^36^.Although we cannot rule out that larger doses may increase alveolar bone mass to a greater extent.

In summary, our study indicates that Scl-Ab can increase alveolar bone mass and mineralization in the *Hyp* mouse model of XLH. While improving alveolar bone quality is likely to have positive effects on the oral health of XLH patients, whether Scl-Ab represents a viable clinical treatment option for XLH patients warrants further study.

## Materials And Methods

### Animals

Female heterozygous (+/*Hyp*, strain 000528) and male wild-type (WT; +/y) mice were purchased from Jackson Laboratory (Bar Harbor, ME, USA). The breeding strategy generated heterozygous (+/*Hyp*) and WT females and hemizygous (*Hyp*/y) and WT males. Mice were weaned at 4 weeks, caged in groups of 3 to 5, maintained on a 12-hour dark/light cycle, and provided standard chow (2018, Teklad) and water ad libitum. All mice were randomly assigned twice weekly 25 mg/kg subcutaneous injections of either Scl-Ab (Amgen Inc, Thousand Oaks, CA and UCB, Brussels, Belgium) or vehicle (saline). The dose was chosen based on previous rodent studies^35^. Injections began at 12 weeks of age and continued for 8 weeks, until sacrifice at 20 weeks of age.

Tissues were collected 24-hours after the last injection. Blood was collected via cardiac puncture and allowed to clot at room temperature for 30 mins before centrifugation at 3,400 rpm for 15 min at 4°C for serum separation. Mandibles were collected in 10% formalin and after 48 hours were separated into left and right hemi-mandibles and stored in 70% ethanol. The total sample size for each group was 12, 12, 10, and 10 for male WT vehicle treated, WT Scl-Ab treated, *Hyp* vehicle treated, and *Hyp* Scl-Ab treated, respectively, and 13, 14, 10, and 12 for female WT vehicle treated, WT Scl-Ab treated, *Hyp* vehicle treated, and Hyp Scl-Ab treated, respectively. The sample size used was based on our tissues available from our published study^14^. All animal studies were approved by the Rush University Institutional Animal Care and Use Committee and were designed to confirm to ARRIVE guidelines. No unexpected adverse events were encountered.

### Micro-computed tomography

Left hemi-mandibles were micro-computed tomography (microCT, μCT50, Scanco Medical). Four separate analyses were performed to characterize alveolar bone, enamel, dentin/cementum complex, and pulp volume. Left hemi-mandibles were microCT scanned while submerged in distilled water. Hemimandibles were placed into a custom designed sample holder with the buccal plane was facing downwards, and images were collected in the sagittal plane. Scanning parameters were 55 kVp and 145 μA, with a 500 ms integration time, a 6μm isotropic voxel size, and a 0.5 mm aluminum filter. Alveolar bone was evaluated on the furcation area between the roots of the first molar, as described elsewhere^37^. A total number of 70 slices were evaluated within the middle of the first molar. The primary outcome of alveolar bone was bone volume per total volume (BV/TV). Enamel, cementum/dentin, and dental pulp regions of interest were identified in the first molar using previously defined thresholding techniques^38^. Enamel, cementum/dentin, and dental pulp regions of interest were identified in the first molar using previously defined thresholding techniques^38^. Enamel was analyzed with a lower threshold of 1600 mg HA/cm^3^ and an upper threshold of 3000 mg HA/cm^3^. Cementum/dentin was analyzed with a lower threshold of 650 mg HA/cm^3^ and an upper threshold of 1600 mg HA/cm^3^. Dental pulp was analyzed with a lower threshold of 500 mg HA/cm^3^ and an upper threshold of 650 mg HA/cm^3^.

### Hematoxylin and Eosin Staining

Right and left hemi-mandibles were washed with deionized water, decalcified in 14% ethylenediaminetetraacetic acid disodium salt dihydrate (pH 7.4) (EDTA, Fisher Scientific) for 5 weeks and dehydrated and embedded in Ribbon Pro paraffin (Thermo Scientific). Sections (5 μm thick) were cut either in the coronal or sagittal planes depending on the analysis, using a Leica RM2255. Sections were stained with hematoxylin and eosin (H&E) to evaluate pre-dentin and acellular cementum thicknesses on the distal root of the first molar from sagittal sections (Nikon Eclipse 80i with Osteomeasure). Cellular cementum area was evaluated on the mesial root of the first molar from coronal sections, as reported by others^6,22^

### Picrosirius Red Staining, Polarized Imaging and CT-FIRE Analysis

Hemi-mandible sections were stained using 0.2% phosphomolybdic acid (Electron Microscopy Sciences). Phosphomolybdic acid was added for 3 min and the slides were subsequently rinsed with water. Sirius Red, 0.1% in saturated picric acid was added to the slides for 90 min, followed by two washes in 0.01 N hydrochloric acid, dehydration, and mounting with Permount (Fisher Chemical Permount Mounting Medium).

Stained sagittal sections were imaged under brightfield light (Nikon Eclipse 80i with Osteomeasure) to quantitate the PDL attachment fraction. The PDL attachment fraction was defined as the total length of acellular cementum along the distal root of the first molar in contact with the PDL, which was subsequently normalized to the total length of the acellular cementum. Slides were also imaged using a under polarized light to visualize collagen fibril orientation (Carl Zeiss AG Axio Observer D1 Inverted Microscope). Polarized images were then evaluated for PDL fiber length, width and angle using CT-FIRE Matlab extension^39^.

### Immunohistochemistry

Sample sections were de-paraffinized and rehydrated. Antigen retrieval was performed by incubating samples in trypsin or sodium citrate (Sigma) at 37°C for 30–45 min. The slides were washed with tris-buffered saline three times followed by blocking in 3% H_2_O_2_ for 10 min at room temperature. Samples were incubated at 4°C overnight in primary antibody for OPN (Invitrogen) at a 1:500 dilution, non-phosphorylated Beta-catenin (Cell Signaling) at a 1:100 dilution, or DMP1 (synthesized in-house, see^40^) at a 1:100 dilution. Anti-Rabbit biotinylated secondary antibody was applied to the samples at a 1:300 dilution for 30 min at room temperature. A tyramide signal amplification kit (Perkin Elmer) was used to amplify the binding signal followed by a DAB Peroxidase Substrate Kit (Vector). Thionin (0.1%) was used as a counterstain for OPN stained tissues, while no counterstaining was used for non-phosphorylated β-catenin and DMP1. Immunohistochemistry staining in the alveolar bone was visualized between the mesial and distal roots of the first molar on coronal sections.

The number of osteocytes within the alveolar bone positively stained with non-phosphorylated β-catenin was quantified by counting the number of positively stained osteocytes and the total number of osteocytes within the alveolar bone (Osteomeasure, OsteoMetrics). All cells were counted at 20x magnification using 5–6 separate images to span the entirety of the alveolar bone between the mesial and distal roots of the first. The percent of positively stained osteocytes was calculated by dividing the number of positively stained osteocytes by the total number of stained and unstained osteocytes and multiplying by 100%. The total sample size was n = 4, 4, 4, 3 for male WT vehicle, WT Scl-Ab, Hyp vehicle, and Hyp Scl-Ab and n = 5, 4, 4, 6 for female WT vehicle, WT Scl-Ab, Hyp vehicle, and Hyp Scl-Ab.

### Statistical Analysis

Quantitative variables were compared separately for males and females using a two-way analysis of variance (ANOVA) with genotype and treatment as the independent factors. When main effects were significant, post-hoc analysis (independent student’s T-test) was performed to compare the effects of the Scl-Ab treatment. A p-value of < 0.05 was considered statistically significant.

## Figures and Tables

**Table 1:** Cementum and pre-dentin parameters from H&E stained sections.

Variable	WT Vehicle	WT Scl-Ab	*Hyp* Vehicle	*Hyp* Scl-Ab	Genotype	Treatment	Interaction
**Males**							
Acellular Cementum Thickness (μm)	5.22 ± 1.78	5.79 ± 1.54	1.94 ± 0.44^b^	1.57 ± 0.45^b^	**< 0.001**	0.852	0.369
Pre-Dentin Thickness (μm)	4.48 ± 1.04	3.85 ± 2.14	12.37 ± 3.89^b^	11.36 4.68^b^	**< 0.001**	0.538	0.887
Cellular Cementum Area (mm^2^)	0.15 ± 0.08	0.17 ± 0.03	0.15 ± 0.01	0.16 ± 0.04	0.827	0.630	0.933
**Females**							
Acellular Cementum Thickness (μm)	5.91 ± 1.91	5.27 ± 1.66	2.80 ± 1.04^b^	1.72 ± 0.63^b^	**< 0.001**	0.147	0.711
Pre-Dentin Thickness (μm)	4.34 ± 2.83	4.51 ± 2.98	7.33 ± 1.55	7.14 ± 2.28	**0.018**	0.992	0.870
Cellular Cementum Area (mm^2^)	0.17 ± 0.08	0.16 ± 0.02	0.13 ± 0.01	0.17 ± 0.08	0.564	0.648	0.281

Sample sizes for each variable are as follows:

Acellular Cementum -- n = 5, 6, 6, 5 for male WT + vehicle, WT + Scl-Ab, Hyp + vehicle, Hyp + Scl-Ab and n = 6, 6, 5, 7 for female WT + vehicle, WT + Scl-Ab, Hyp + vehicle, Hyp + Scl-Ab

Pre-Dentin Thickness -- n = 6, 7, 6, 3 for male WT + vehicle, WT + Scl-Ab, Hyp + vehicle, Hyp + Scl-Ab and n = 6, 5. 3, 3 for female WT + vehicle, WT + Scl-Ab, Hyp + vehicle, Hyp + Scl-Ab

Cellular Cementum Area -- n = 3, 4, 3, 3 for male WT + vehicle, WT + Scl-Ab, Hyp + vehicle, Hyp + Scl-Ab and n = 4, 5, 5, 3 for female WT + vehicle, WT + Scl-Ab, Hyp + vehicle, Hyp + Scl-Ab

a Indicates significant differences between vehicle and Scl-Ab treated mice of the same genotype.

b Indicates significant differences from vehicle treated WT mice.

**Table 2:** Periodontal ligament (PDL) parameters from picrosirius red stained sections

Variable	WT Vehicle	WT Scl-Ab	*Hyp* Vehicle	*Hyp* Scl-Ab	Genotype	Treatment	Interaction
**Males**							
PDL Attachment Fraction (%)	97.78 ± 1.1	97.83 ± 3.1	31.3 ± 11.6^b^	40.06 ± 22.4^b^	**< 0.001**	0.487	0.492
PDL Fiber Angle (°)	84.0 ± 8.5	90.0 ± 11.4	86.3 ± 14.0	85.3 ± 19.0	0.818	0.636	0.514
PDL Fiber Length (μm)	19.1 ± 2.8	21.0 ± 2.1	19.8 ± 4.2	19.3 ± 1.2	0.675	0.541	0.301
PDL Fiber Width (μm)	2.1 ± 0.1	2.1 ± 0.2	2.2 ± 0.1	2.1 ± 0.1	0.817	0.908	0.400
**Females**							
Periodontal Ligament Attachment Fraction (%)	96.58 ± 4.8	96.86 ± 2.1	53.53 ± 32.6^b^	70.56 ± 16.2^b^	**< 0.001**	0.243	0.258
PDL Fiber Angle (°)	85.1 ± 31.9	75.0 ± 29.4	104.5 ± 21.1	89.0 ± 28.4	0.148	0.264	0.058
PDL Fiber Length (μm)	22.1 ± 3.2	21.2 ± 2.5	21.3 ± 2.6	19.8 ± 2.8	0.325	0.269	0.067
PDL Fiber Width (μm)	2.1 ± 0.1	2.1 ± 0.1	2.2 ± 0.2	2.2 ± 0.1	0.125	0.698	0.608

Sample sizes are as follows:

n = 4, 4, 5, 4 for male WT + vehicle, WT + Scl-Ab, Hyp + vehicle, Hyp + Scl-Ab and n = 7, 5, 4, 5 for female WT + vehicle, WT + Scl-Ab, Hyp + vehicle, Hyp + Scl-Ab

a Indicates significant differences between vehicle and Scl-Ab treated mice of the same genotype.

b Indicates significant differences from vehicle treated WT mice.

## Data Availability

All data associated with this study are presented in the paper.
